# Magnetic resonance tumor regression grade (MR-TRG) to assess pathological complete response following neoadjuvant radiochemotherapy in locally advanced rectal cancer

**DOI:** 10.18632/oncotarget.21778

**Published:** 2017-10-10

**Authors:** Marco Rengo, Simona Picchia, Simona Marzi, Davide Bellini, Damiano Caruso, Mauro Caterino, Maria Ciolina, Domenico De Santis, Daniela Musio, Vincenzo Tombolini, Andrea Laghi

**Affiliations:** ^1^ Department of Radiological Sciences, Oncology and Pathology. “Sapienza” - University of Rome, Diagnostic Imaging Unit - I.C.O.T. Hospital, Latina, Italy; ^2^ Medical Physics Laboratory, Regina Elena National Cancer Institute, Rome, Italy; ^3^ Department of Radiological Sciences, Oncology and Pathology. “Sapienza” - University of Rome, Radiotherapy Unit, Policlinico Umberto I, Rome, Italy; ^4^ Radiology Unit, Regina Elena National Cancer Institute, Rome, Italy

**Keywords:** rectal cancer, neoadjuvant therapy, magnetic resonance imaging, prognosis

## Abstract

This study aims to evaluate the feasibility of a magnetic resonance (MR) automatic method for quantitative assessment of the percentage of fibrosis developed within locally advanced rectal cancers (LARC) after neoadjuvant radiochemotherapy (RCT). A total of 65 patients were enrolled in the study and MR studies were performed on 3.0 Tesla scanner; patients were followed-up for 30 months. The percentage of fibrosis was quantified on T2-weighted images, using automatic K-Means clustering algorithm. According to the percentage of fibrosis, an optimal cut-off point for separating patients into favorable and unfavorable pathologic response groups was identified by ROC analysis and tumor regression grade (MR-TRG) classes were determined and compared to histopathologic TRG. An optimal cut-off point of 81% of fibrosis was identified to differentiate between favorable and unfavorable pathologic response groups resulting in a sensitivity of 78.26% and a specificity of 97.62% for the identification of complete responders (CRs). Interobserver agreement was good (0.85). The agreement between P-TRG and MR-TRG was excellent (0.923). Significant differences in terms of overall survival (OS) and disease free survival (DFS) were found between favorable and unfavorable pathologic response groups. The automatic quantification of fibrosis determined by MR is feasible and reproducible.

## INTRODUCTION

Magnetic Resonance (MR) is the most accurate imaging modality to stage locally advanced rectal cancer (LARC). MR role in stratification of patient risk and in guiding patient management has been widely investigated [1–6]. The strength of this technique is based on its ability to distinguish normal rectal wall from pathologic tissues on the basis of the differences in signal intensity achievable on T2-weighted sequences [4].

Neoadjuvant radiochemotherapy (RCT), which is the treatment of choice in patients with locally advanced rectal cancer (LARC) [7, 8], induces a development of fibrosis within the tumor which decreases the contrast with vital tissue. Thus, the use of MR for restaging after RCT is hampered by the difficulty to distinguish post-treatment fibrosis from residual tumor, due to their very similar T2 signal intensity [9].

Identification of complete responders (CR) after RCT is crucial because there is an accumulating evidence that in these patients, surgery may be deferred and an active surveillance can be performed [10, 11]. There is no consensus on the method to identify CR after RCT [12].

Histopathological tumor regression grade (P-TRG), defined as the ratio between fibrosis and residual tumor, is routinely used to assess response to therapy and demonstrated to be an important predictor of patient’s outcome [13–16].

Previous experiences [17–19] developed a TRG system based on MR T2 weighted sequences by applying the principles of histopathological grading, MR-TRG, and demonstrated a good correlation with patient’s outcome. However, in all published experiences, the quantification of fibrosis was assessed on the basis of a visual evaluation performed by experienced radiologists, reporting variable results [17–19].

The primary aim of our study was to develop an algorithm for automatic quantification of the fibrosis induced by RCT and to evaluate whether it can be used to identify CRs. The secondary aim of the study was to use the quantitative evaluation of fibrosis to develop an MR-TRG score and to evaluate the agreement with P-TRG.

## RESULTS

**Figure 1:**
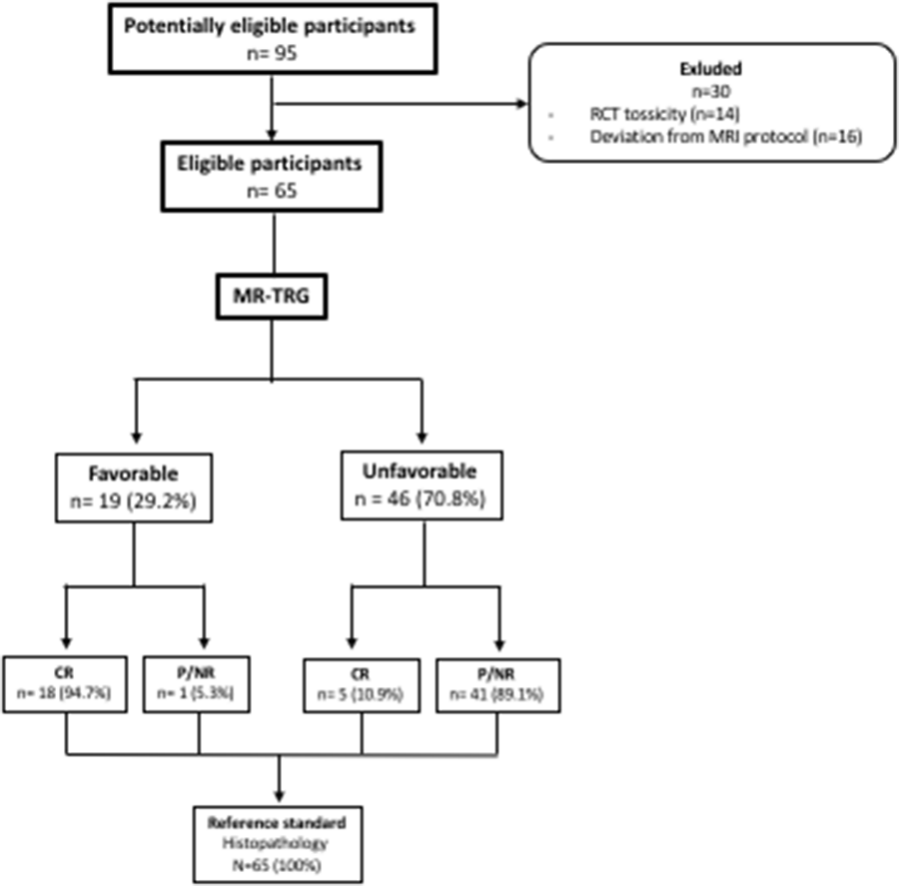
Study population Flow chart detailing the patient selection process.

A total of 65 patients completed all the three phases of the study (Figure 1). Twenty-three patients (35.3%) achieved complete response (pCR) at histology, while 42 patients (64.7%) achieved either partial response (pPR) or no response (pNR). No differences in terms of sex, age or tumor characteristics were observed between pCR and pP/NR. Patients characteristics are summarized in Table 1.

**Table 1:** Baseline characteristics of patient’s population

*Characteristics*	*Total*	*pCR*	*pNR/pPR*	*P-value*
***Sex***				0.65
*Male*	44(66.7%)	14 (60.9%)	29 (69.1%)	
*Female*	21 (33.3%)	9 (39.1%)	13 (30.9%)	
***Age y (±SD)***	64.8 (±8.43)	62.5 (±6.7)	65.3 (±9.2)	
***T stage***				0.78
*2*	1 (1.5%)	1 (4.3%)	1 (2.3%)	
*3*	56 (86.2%)	19 (82.6%)	36 (85.7%)	
*4*	8 (12.3%)	3 (13.1%)	5 (12%)	
***N stage***				0.15
*0*	12 (18.4%)	5 (21.7%)	8 (19.1%)	
*1*	26 (40%)	6 (26.1%)	18 (42.8%)	
*2*	27 (41.6%)	12 (52.2%)	16 (38.1%)	
***Overall stage***				0.26
*II*	12 (18.4%)	5 (21.7%)	8 (19.1%)	
*IIIA*	1 (1.5%)	0 (0%)	1 (2.3%)	
*IIIB*	27 (41.6%)	7 (30.4%)	19 (45.3%)	
*IIIC*	25 (38.5%)	11 (47.9%)	14 (33.3%)	
***Tumor dimension***				0.23
*≤ 5 cm*	43 (66.2%)	18 (78.3%)	26 (61.9%)	
*> 5 cm*	22 (33.8%)	5 (21.7%)	16 (38.1%)	
***Distance from anal verge***				0.32
*≤ 5 cm*	37 (56.9%)	14 (60.9%)	23 (54.7%)	
*>5 ≤ 8 cm*	15 (23.1%)	2 (8.7%)	11 (26.2%)	
*> 8 cm*	13 (20%)	7 (30.4%)	8 (19.1%)	

MR acquisitions were performed between 38 and 48 days (mean: 45.66, median: 43.44, SD: ±1.58) after the end of neoadjuvant RCT and between 4 and 7 days (mean: 5.02, median: 4.95, SD±0.76) before surgery.

Mean time consumed for contouring the entire tumor volume was 10 minutes (± 3.62 minutes, median: 9.5 minutes).

According to ROC analysis, a cut-off value of 81% of fibrosis was identified to discriminate between favorable and unfavorable pathologic response groups. Thus, in the group of favorable pathologic response were included only tumors with a percentage of fibrosis equal or greater than 81%. Accordingly, in the group of unfavorable pathologic response all tumors with a percentage of fibrosis equal or lower than 80% were included.

**Figure 2:**
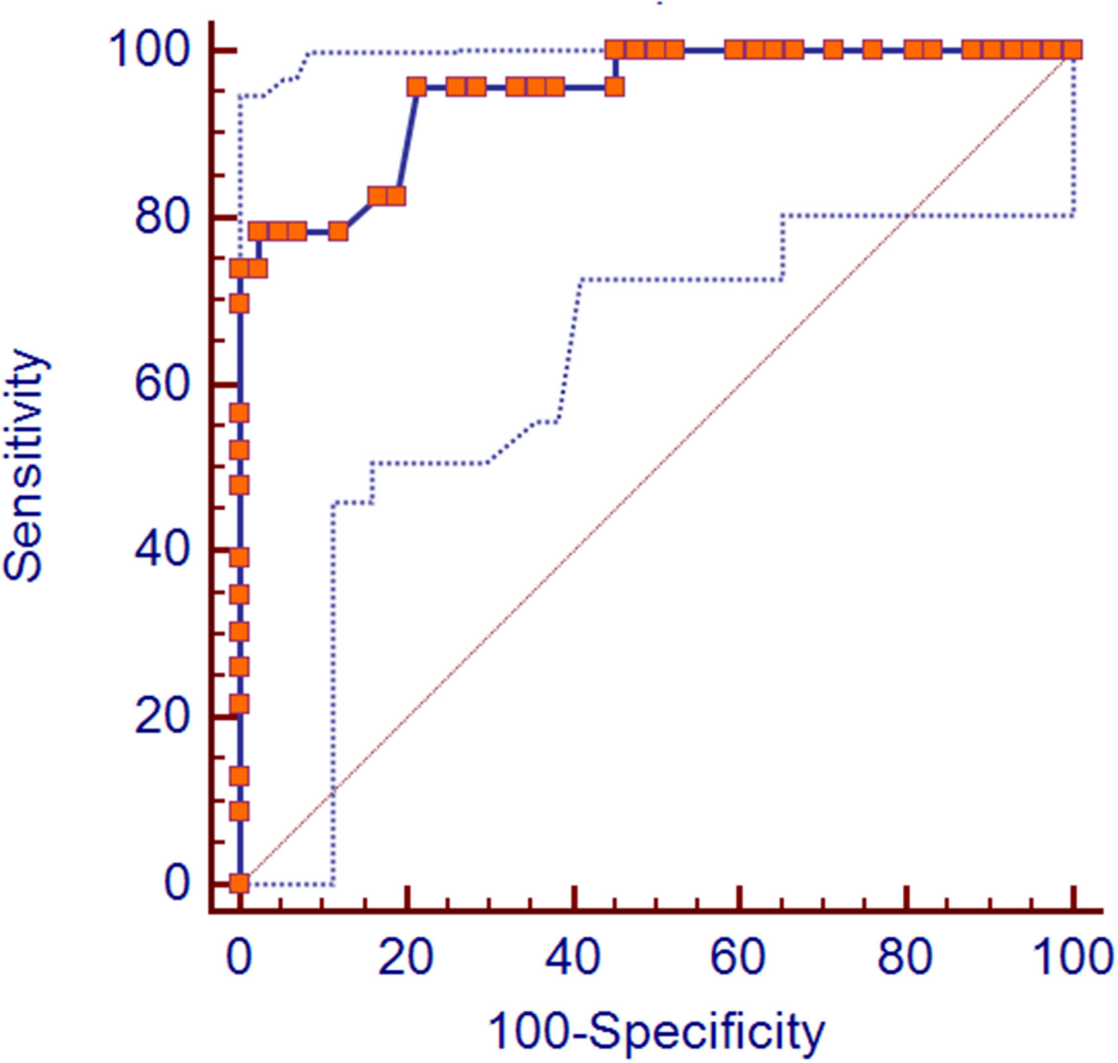
ROC curves The figure shows the Area Under the Curve (AUC) of the Receiver Operating Characteristic (ROC) Curve Analysis with 95% Confidence Limits (AUC = 0.947 and CI: 0.861 - 0.987).

Performances of the automatic quantification of fibrosis algorithm are summarized in Table 2. Using the aforementioned cut-off value of fibrosis, a sensitivity of 78.26% (95% CI: 56.3 – 92.5) and specificity of 97.62% (95% CI: 87.4 – 99.9) were calculated (Figure 2). Eighteen of the 23 pCRs (78%) were included in the favorable group. Five pCRs (22%) were included in the unfavorable groups. One pP/NR (2%) was included in the favorable group.

**Table 2:** Accuracy for the detection of CRs

		*95% CI*	*P value*
AUC	0.947	0.861 to 0.987	<0.0001
sensitivity	78.26	56.3 – 92.5	
specificity	97.62	87.4 – 99.9	
PPV^*^	94.7	74.0 – 99.9	
NPV^**^	89.1	76.2 – 96.4	

^*^ Positive predictive value; ^**^ negative predictive value.

According to ROC analysis a lower cut-off value of 62% of fibrosis corresponds to a sensitivity of 100% (95% CI: 85.2 – 100) and a specificity of 54.76% (95% CI: 38.7 – 70.2) while a higher cut-off value of 83% of fibrosis corresponds to a sensitivity of 73.9% (95% CI: 51.6 – 89.8) and a specificity of 100% (95% CI: 91.6 – 100).

During the follow-up period (30 months), 14 patients (21.5%) died as a result of cancer-related causes. Twenty-five (38%) patients had disease progression for local recurrences with or without metastatic disease.

**Figure 3:**
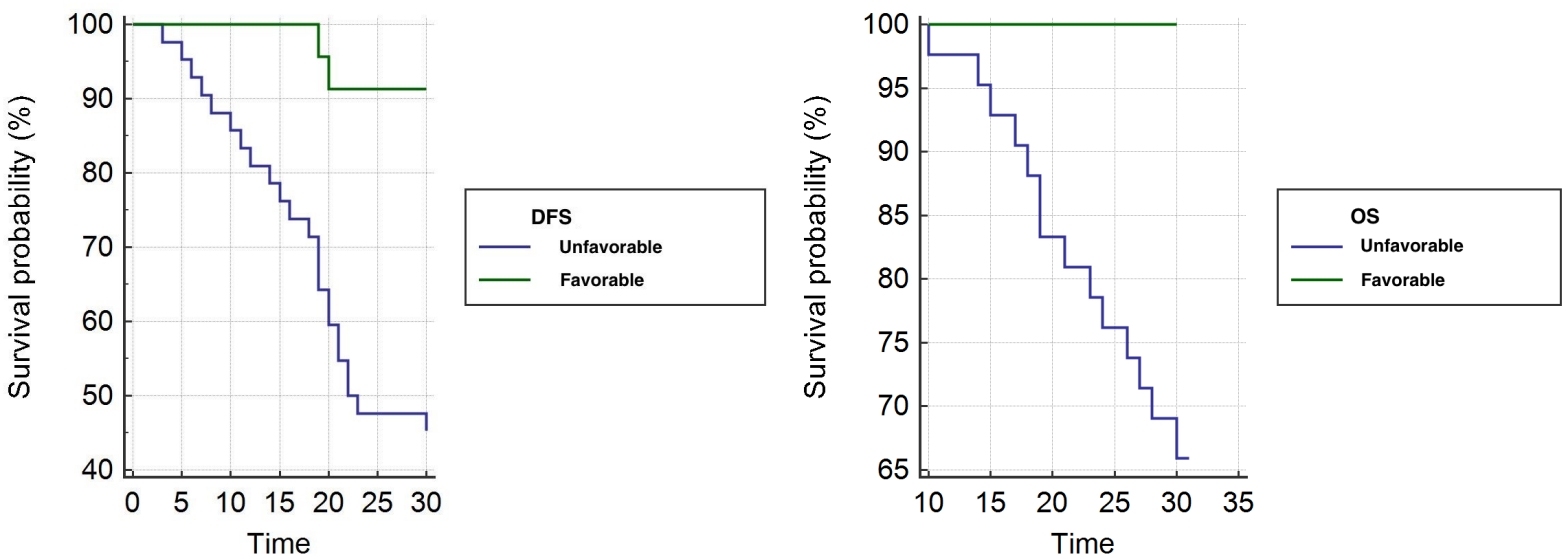
Kaplan Meier curves Kaplan-Meier curves of DFS (a) and OS (b) for unfavorable and favorable group.

A significant difference between favorable and unfavorable pathologic response groups for OS and DFS was observed; hazard ratios were 5.31 (95% CI, 1.81 to 15.55) and 4.20 (95% CI, 1.88 to 9.39) respectively. Kaplan-Meier survival curves are shown in Figure 3.

Median OS time at 30 months for patients with unfavorable pathologic response was 27.58 months (95% CI, 25.83 to 29.34) compared with 30 months (95% CI, 30 to 30) for patients with favorable pathologic response (P=0.0023).

Median DFS at 30 months for patients with unfavorable pathologic response was 22.16 months (95% CI, 19.47 to 24.85) compared with 29.08 months (95% CI, 27.87 to 30.29) for patients with favorable pathologic response (P=0.0005).

The interobserver agreement for the manual contouring and quantification of the tumor volume was moderate (ICC=0.48; 95% CI, 0.26 to 0.76; P=0.0216) while the one for the automatic quantification of the percentage of fibrosis was very good (ICC=0.89; 95% CI, 0.83 to 0.93; P<0.0001).

The agreement between MR-TRG and P-TRG classes was very good (weighted kappa = 0.91; 95% CI = 0.85 – 0.94; P<0.0001).

## DISCUSSION

Our results demonstrated that the automatic fibrosis quantification is feasible and reproducible. The proposed method provided high sensitivity and specificity for the identification of CR after neoadjuvant RCT. The automatic fibrosis quantification was able to identify CR and P/NR, with high sensitivity (78%), specificity (97%) and AUC (0.947). These results should be compared to previous studies using a visual assessment and histology as reference standard. Bhoday et al [19] correctly identified 17 out of 18 CRs in a total population of 143 patients using a cut-off value of 50% of fibrosis to classify favorable MR-TRG group. However, using this cut-off value a large number of false positives were observed resulting in an overall sensitivity of 15.3% and a specificity of 96.9%. Patel et al [6], using the same visual approach of Bhoday et al, reported a sensitivity of 61.7% and a specificity of 90.9% for identification of ypT0-T3a tumors considered as favorable result after neoadjuvant RCT. Performances of our software-based method are notable not only in terms of sensitivity and specificity, but especially because in our cohort only pT0 tumors were considered as CRs.

Other MRI biomarkers have been proposed for identification of CRs. In particular, diffusion weighted imaging (DWI) and dynamic contrast-enhanced MRI (DCE-MRI) showed the highest accuracy. A recent study reported a sensitivity of 35% and a specificity of 94% [25] combining DWI and T2w morphologic sequences. It has been demonstrated that the evaluation of DWI volumes increases the sensitivity of this biomarker up to 64% [26]. Several studies investigated the accuracy of DCE-MRI reporting variable results. However, the evaluation of standardized index of shape (SIS), demonstrated to be the most accurate and reproducible method to identify CRs with a sensitivity higher than 90% and a specificity higher than 80% [27, 28].

In the era of organ preservation strategies, it is crucial to correctly identify CRs, needing a high sensitivity, but it is even more important to correctly classify P/NR, needing a high specificity, to avoid the delay of surgery in a patient with residual tumor.

Because the automatic fibrosis quantification can be used to determine MR-TRG classes, a comparison with P-TRG should be performed. We found a good correlation between the two methods (weighted kappa: 0.91), as opposite to a previously published study [29]. One of the possible explanation of the good results obtained is the use of 3.0 Tesla MRI scanner while most of the previous literature is based on datasets acquired on 1.5 Tesla systems. The higher strength of the magnetic field is, in fact, associated with a higher signal and overall higher image quality.

Despite our good results, a limitation of our approach should be underlined. The algorithm we used divides pixels necessarily into two groups: high signal for residual tumor and low signal for fibrosis. With this approach, MR-TRG 0 and 5 (respectively 100% and 0% of fibrosis) cannot be calculated, thus we grouped p-TRG 0 and 1 as well as p-TRG 4 and 5 and we finally compared four TRG classes for both p-TRG and MR-TRG. However, a real site-to-site comparison between the two methods is difficult to be realized in clinical practice, since it would require the surgical specimen to maintain the *in vivo* anatomy (as in MRI) while it is preliminarily dissected and fixed in formalin. Another major difference is that the evaluation with MRI is performed on the entire tumor volume while histopathology is performed on sample sections.

Histopathologic TRG has been shown to be an independent prognostic factor after neoadjuvant RCT [30, 31]. And studies using MR-TRG demonstrated similar data, showing a significant difference in terms of OS and DFS between poor and good MR-TRG groups [6, 32, 33]. Also, our results found a significant difference in terms of OS and DFS between favorable and unfavorable pathologic response groups determined with our algorithm.

One of the main limits of the visual fibrosis quantification is the poor interobserver agreement. The reproducibility of the method has been previously investigated reporting a variable agreement between readers, ranging between poor and good [18, 29, 34]. In these previous publications, a higher agreement was observed for identification of poor responders compared with good responders. Moreover, Patel et al [18] found disagreement between central reviewer and second reviewer (expert reader and less experienced reader, respectively) in the identification of complete responders, due to a higher percentage of good responders assessed by the central reviewer, this result demonstrated the influence of experience in visual assessment. In our study, to reduce potential bias related to reader expertise, we used low experienced readers (fifth year residents in radiology), and we found a moderate interobserver agreement (ICC: 0.48) for the manual quantification of the tumor volume which is an operator dependent process. However, the interobserver agreement for the automatic fibrosis percentage quantification, based on the datasets manually contoured by the operators, returned very good (ICC=0.89). This result underlines the usefulness of an automatic quantification method, which is crucial to standardize the procedure.

The time consumed to draw the ROI around the margins of the tumor on all axial slices, consisting in 10 minutes per dataset, should be considered as a limit of the proposed method. However, despite this drawback, the computing system we used only takes few seconds to perform the analysis.

Despite the excellent performances of the proposed method, we should underline some limitations. First, the quantification of the percentage of fibrosis was a retrospective process performed on a prospectively recruited population. Second, the study population is relatively small. Nevertheless, our sample size is similar to the ones reported in most of the previous studies. Third, we did not validate the method on a control group. Finally, we applied our approach only to T2 weighted images. Theoretically, the algorithm we used also works with other sequences like DWI or perfusion maps. However, this would require a dedicated study.

In conclusion, our results demonstrated that automatic fibrosis quantification with MRI is feasible, provides better results compared to visual assessment and can be considered a reliable method to identify CRs after neoadjuvant RCT.

## MATERIALS AND METHODS

### Subjects

The study was conducted according to Good Clinical Practice (GCP)-International Conference on Harmonization (ICH) [20]. All patients signed a written informed consent to be enrolled in the study. The protocol was approved by the Local Ethical Committee (Rif. 2737/28.03.2013).

All patients were enrolled between May 2013 and December 2014.

Patients with histologically-confirmed rectal adenocarcinoma (Stage II and Stage III according to the International Union Against Cancer (IUCC) classification [21]), were included in this non-randomized, prospective, multi-center trial (two centers). Exclusion criteria are listed in Table 3.

**Table 3:** Exclusion criteria

Evidence of contraindications to MR examination (e.g. pacemaker, cochlear implant, etc.).
Incomplete MR acquisition or histopathological analysis.
Contraindication to the use of neoadjuvant therapy or surgical treatment.
Suspension of neoadjuvant combination chemotherapy-radiation treatment prior to surgery, presence of synchronous tumors, mucinous histotype, neurological or psychiatric disorders or previous pelvic radiotherapy.
Hypersensitivity to the study drug or to one of the excipients.
Legal incapacity.
Concurrent treatment with experimental drugs or participation in another clinical trial with any investigational drug within 30 days before study screening.
Alcohol or drug abuse.

All patients underwent optical colonoscopy with biopsy for immunohistochemical analysis and an MR study for tumor staging. Two weeks after staging, patients started the neoadjuvant RCT protocol. An MR study for restaging after RCT was performed within one week before surgery. All patients underwent total mesorectal excision (TME) 6-8 weeks after the end of RCT. All gross specimens were analyzed by one single pathologist. Patients were followed up for thirty months at intervals of three months with physical examination, routine blood tests and yearly whole body computed tomography to assess local recurrences or distant metastases.

### MR protocol and image analysis

All MR examinations were performed using a 3T scanner (Discovery MR750, General Electrics, Milwaukee, Wisconsin, USA) using a phased-array coil, with the protocol described in Table 4.

**Table 4:** MR protocol

Sequence	Planes	TR/TE (msec)	NEX	Matrix	Slice thickness (mm)	Other
2D FRFSE^*^ T2w	orthogonal and parallel to the long axis of the tumor	4172 / 122.3	2	512×512	4	
2D SSEPI^**^ DWI	Axial	4400 / 81.4		256×256	4	B values: 0, 10, 20, 30, 50, 60, 100, 200, 600, 800 and 1000 sec/mm2
3D FSPGR^***^ T1w	Axial	13.6 / 3.3	2	512×512	4	FA: 15°
3D FSPGR^***^ T1w(perfusion imaging)	Axial	13.6 / 3.3	2	512×512	4	IV administration of 2ml/kg of body weight of gadolinium chelate followed by a 15 ml saline flush at a rate of 2 ml/s.

^*^ Fast Relaxation Fast Spin Echo; ^**^ Single-Shot Echo Planar Imaging; ^***^ Fast Spoiled Gradient-Echo.

For the purpose of this study, only T2 weighted images acquired after neoadjuvant RCT were analyzed.

**Figure 4:**
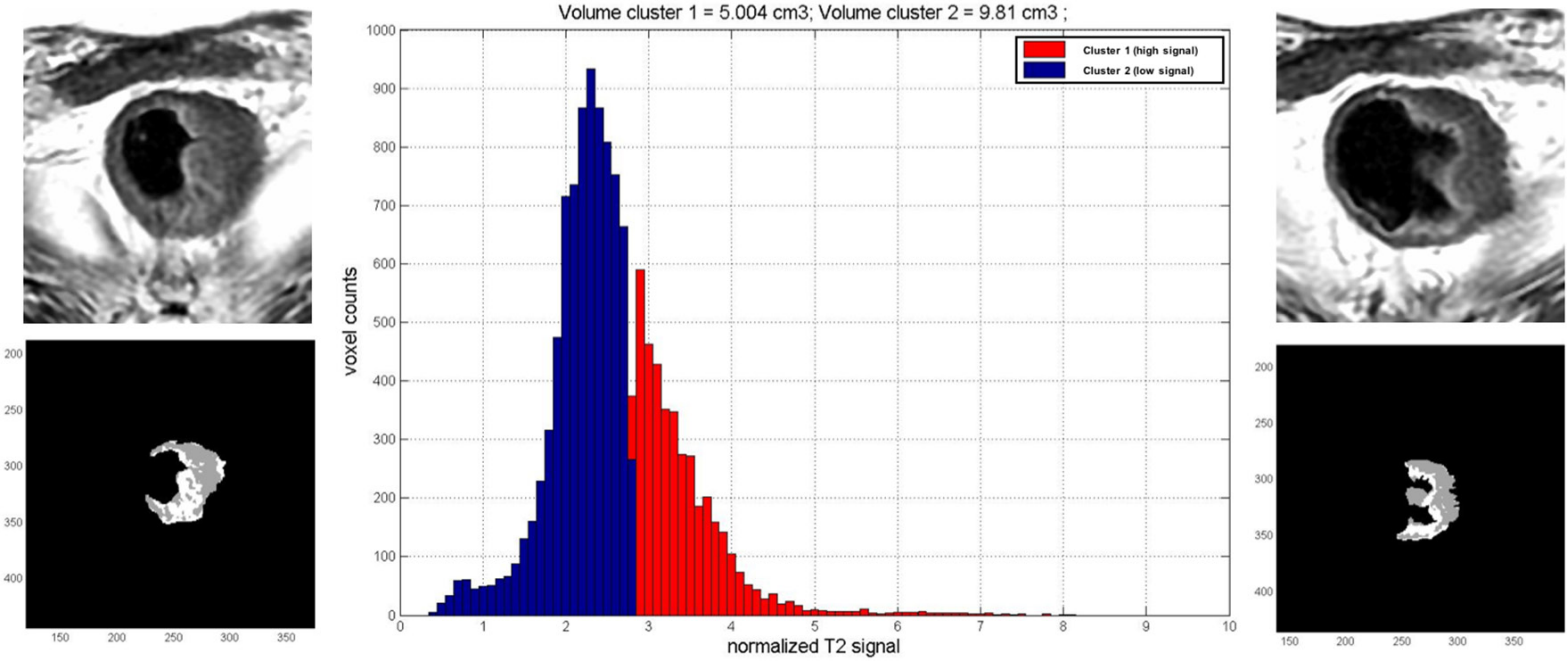
Quantification of fibrosis percentage This example shows the native T2w images and the corresponding one elaborated with our software. The gray pixels represent the low signal intensity (fibrosis) and the white ones the high signal intensity (residual tumor). The histogram shows the distribution of pixels on the basis of their signal intensity.

Following a previous experience [22], we decided to use the algorithm K-means for the automatic quantification of fibrosis. This algorithm, implemented in an in-house software developed in MATLAB software (The MathWorks Inc., Natick, Massachusetts, United States), automatically partitions data (n) into *k* numbers of mutually exclusive clusters; (*k*<n). The number of partitions is driven by the operator. We set *k* = 2 to cluster pixels into two partitions on the basis of their median signal intensity: a high signal intensity partition assumed to represent the residual tumor and a low signal intensity partition assumed to represent fibrosis (Figure 4).

Before the automatic analysis, a manual contouring of the entire tumor volume was performed on each axial section of T2-weighted images.

Tumor volume was considered as the entire mass appreciable including both fibrosis and vital tissue and excluding the lumen of the colon.

Two fifth year residents in radiology (with 1-year of experience on MR of the rectum), separately, manually contoured each lesion with *3D-slicer*, a free platform for biomedical research (Brigham and Women’s Hospital, Boston, MA, USA). Time consumed to contour the entire tumor volume was recorded.

The manual contouring process provided as output the total tumor volume in cubic millimeters. The contoured dataset was processed with the k-means algorithm which provided two outputs, the volumes of low (fibrosis) and high (residual tumor) signal intensity pixels in cubic millimeters.

The percentage of fibrosis within the total tumor volume was calculated according to the following formula:

(Fibrosis volume / Tumor volume) × 100

Then, we divided tumors into four classes, according to the P-TRG described by Dworak-Rodel, on the basis of the percentage of fibrosis developed after RCT. Thus, in MR-TRG class 1 were grouped all tumors with ≤ 25% of fibrosis, in class 2 between 26% and 50%, in class 3 between 51% and 75% and in class 4 ≥ 76%.

### P-TRG Assessment

A pathologist, blinded to MR and biopsy findings, analyzed in random order all the gross specimens. The rectal segment harboring the neoplasm was examined by sectioning orthogonal to the long axis, obtaining 2-3 mm thick macro section specimens. According to Dowrak-Rodel technique tumor regression was semi-quantitatively assessed by the amount of viable tissue versus the amount of fibrosis, ranging from no evidence of fibrosis to a complete response with no residual tumor identifiable, [23].

### Neoadjuvant radiochemotherapy

Radiochemotherapy was administered following the standard of care in our hospital [24]. Radiation therapy was performed with a fractioned 3D-conformational technique (45 Gy in 5 weeks) to the whole pelvis. An additional dose of 5.4-9 Gy was administered to the tumor volume in 3-5 days (6-15 MV energy photons).

Chemotherapy was administered through a central venous access (port-a-cath) as follows: 5 or 6 cycles of oxaliplatin (2-hour infusion 50 mg/m^2^) the first day of each week of radiotherapy followed by five daily continuous infusions of 5-FU 200 mg/m^2^/die.

Oxaliplatin infusion was preceded by desamethasone (8 mg) and ondansetron (8 mg) administration. Toxicity was evaluated according to NCI-CTC version 3.0dsds.

### Statistical analysis

All continues variables were expressed as median and mean ± standard deviation (SD).

The percentage of fibrosis developed after RCT was automatically determined by the K-means algorithm. An optimal cut-off value of percentage of fibrosis for separating patients into favorable and unfavorable pathologic response groups was identified by receiver operating characteristics (ROC) analysis, with Youden index. This was done plotting the percentage of fibrosis as absolute value and the result of histology, dichotomized as complete responder (CR) or partial/non-responder (P/NR).

Diagnostic performance for the identification of CR was calculated by means of ROC curves. The area under the curve (AUC), sensitivity (SE), specificity (SP), positive predictive value (PPV) and negative predictive value (NPV) were calculated.

Differences in terms of overall survival (OS) and disease free survival (DFS) between favorable and unfavorable pathologic response groups were calculated by using Kaplan-Meier product limit method with univariate log-rank test.

Since the process includes two steps, consisting of manual contouring of the tumor and the automatic quantification of the percentage of fibrosis, the reproducibility was evaluated for each step by means of intraclass correlation coefficient (ICC).

Weighted Kappa statistic was performed to evaluate the agreement between MR-TRG and P-TRG classes.

Statistical analyses were carried out using a commercially available statistical software (MedCalc Statistical Software version 16.4.3, MedCalc Software bvba, Ostend, Belgium and GraphPad Prism version 5.0, GraphPad Software, La Jolla, California, USA). A two-tailed *P* < 0.05 was considered statistically significant.

## Abbreviations

MR-TRG: Magnetic Resonance Tumor Regression Grade
P-TRG: pathological TRG
CR: complete responders
P/NR: partial/non-responders
pCR: pathological complete responders
pP/NR: pathological partial/non-responders
RCT: radiochemotherapy
LARC: locally advanced rectal cancer
ICC: interclass correlation coefficient analysis
AUC: area under the curve
SE: sensitivity
SP: specificity
PPV: positive predictive value
NPV: negative predictive value
OS: overall survival
DFS: disease free survival
TME: total mesorectal excision
AIRC: Association for Cancer Research
GCP-ICH: Good Clinical Practice-International Conference on Harmonization
